# A Survey on Swarming With Micro Air Vehicles: Fundamental Challenges and Constraints

**DOI:** 10.3389/frobt.2020.00018

**Published:** 2020-02-25

**Authors:** Mario Coppola, Kimberly N. McGuire, Christophe De Wagter, Guido C. H. E. de Croon

**Affiliations:** ^1^Micro Air Vehicle Laboratory (MAVLab), Department of Control and Simulation, Faculty of Aerospace Engineering, Delft University of Technology, Delft, Netherlands; ^2^Department of Space Systems Engineering, Faculty of Aerospace Engineering, Delft University of Technology, Delft, Netherlands

**Keywords:** swarm, challenges, review, robustness, autonomous, micro air vehicles, drones, MAV

## Abstract

This work presents a review and discussion of the challenges that must be solved in order to successfully develop swarms of Micro Air Vehicles (MAVs) for real world operations. From the discussion, we extract constraints and links that relate the local level MAV capabilities to the global operations of the swarm. These should be taken into account when designing swarm behaviors in order to maximize the utility of the group. At the lowest level, each MAV should operate safely. Robustness is often hailed as a pillar of swarm robotics, and a minimum level of local reliability is needed for it to propagate to the global level. An MAV must be capable of autonomous navigation within an environment with sufficient trustworthiness before the system can be scaled up. Once the operations of the single MAV are sufficiently secured for a task, the subsequent challenge is to allow the MAVs to sense one another within a neighborhood of interest. Relative localization of neighbors is a fundamental part of self-organizing robotic systems, enabling behaviors ranging from basic relative collision avoidance to higher level coordination. This ability, at times taken for granted, also must be sufficiently reliable. Moreover, herein lies a constraint: the design choice of the relative localization sensor has a direct link to the behaviors that the swarm can (and should) perform. Vision-based systems, for instance, force MAVs to fly within the field of view of their camera. Range or communication-based solutions, alternatively, provide omni-directional relative localization, yet can be victim to unobservable conditions under certain flight behaviors, such as parallel flight, and require constant relative excitation. At the swarm level, the final outcome is thus intrinsically influenced by the on-board abilities and sensors of the individual. The real-world behavior and operations of an MAV swarm intrinsically follow in a bottom-up fashion as a result of the local level limitations in cognition, relative knowledge, communication, power, and safety. Taking these local limitations into account when designing a global swarm behavior is key in order to take full advantage of the system, enabling local limitations to become true strengths of the swarm.

## 1. Introduction

Micro Air Vehicles (MAVs), or “small drones,” are becoming commonplace in the modern world. The term refers to small, light-weight, flying robots. Several MAV designs exist, including multirotors (Kumar and Michael, 2012), flapping wing (Michelson and Reece, 1998; Wood et al., 2013; de Croon et al., 2016), fixed wing (Green and Oh, 2006), morphing designs (Falanga et al., 2019b), or “hybrid” vehicles (Itasse et al., 2011). Of these, quadrotors have enjoyed the spotlight due to their high maneuverability, their ability to take-off vertically (as opposed to most fixed wing MAVs, for instance), and their relative simplicity in design (Gupte et al., 2012; Kumar and Michael, 2012). MAVs can be used for surveillance and mapping (Mohr and Fitzpatrick, 2008; Scaramuzza et al., 2014; Saska et al., 2016b), infrastructure inspection (Sa and Corke, 2014), load transport and delivery (Palunko et al., 2012), or construction (Lindsey et al., 2012; Augugliaro et al., 2014). Such applications are particularly useful in areas that are not easily accessible by humans, like forests or disaster sites (Alexis et al., 2009; Achtelik et al., 2012). Smaller and lighter designs push the boundaries of their applications further. Aside from the asset of increased portability, smaller MAVs can also navigate through tighter spaces, such as narrow indoor environments with higher agility (Mohr and Fitzpatrick, 2008). They also cause less damage to their surroundings (including people) in the event of a collision, making them intrinsically safer tools (Kushleyev et al., 2013).

Unfortunately, smaller size comes at the expense of more limited capabilities. The interplay between limited flight time, limited sensing, and limited power hinder an MAV from performing grander tasks on its own. This has created a strong interest in developing MAV *swarms* (Yang et al., 2018). The paradigm of swarm robotics aims to transcend the limitations of a single robot by enabling cooperation in larger teams. This is inspired by the animal kingdom, where animals and insects have been observed to unite forces toward a common goal that is otherwise too complex or challenging for the lone individual (Garnier et al., 2007). Using several robots at once can bring several advantages and possibilities, such as: redundancy, faster task completion due to parallelization, or the execution of collaborative tasks (Martinoli and Easton, 2003; Trianni and Campo, 2015; Nedjah and Junior, 2019). The control of robotic swarms is envisioned to be fully distributed. The individual robots perceive and process their environment locally and then act accordingly without global awareness or direct awareness of the final goal of the swarm. Nevertheless, by means of collaboration, the robots can achieve an objective that they would not have been able to achieve by themselves. As they say: there is strength in numbers.

It is easy to imagine swarms of MAVs jointly carrying a load that is too heavy for a single one to lift, or persistently exploring an area without interruption. As is often the case, however, putting such visions into practice is another story altogether. Developing self-organizing swarms of MAVs in the real world is a multi-disciplinary challenge coarsely divided in two main aspects. One aspect is that of the individual MAV design, where the local abilities of a single MAV are defined. The second aspect is the swarm design, whereby we need to develop controllers with which the global goal can be efficiently achieved, autonomously, by the swarm. To make matters more complicated, the two are not decoupled. As we shall explore in this paper, there exist fundamental links between the local limitations of an MAV and the behaviors that a swarm of MAVs could, or should, execute as a result. Vice versa, in order to realize certain swarm behaviors, there are local requirements that the individual MAVs must meet. This bond between the local and the global cannot be ignored if MAV swarms are to be brought to the real world. In this paper, we aim to reconcile these two aspects and present a discussion of the fundamental challenges and constraints linking local MAV properties and global swarm behaviors.

## 2. Co-dependence of Swarm Design and Individual MAV Design

Let us begin from the primary challenge of swarm robotics: to design local controllers that successfully lead to global swarm behaviors (Şahin et al., 2008). Concerning MAVs, these global behaviors include, but are not limited to: collaborative transport, collaborative construction, distributed sensing, collaborative object manipulation, and parallelized exploration and mapping of environments. Albeit the individual MAV may be limited in its ability to successfully perform these tasks (for instance, as areas get larger or loads get heavier), they can be tackled by collaborating in a swarm. Generally, swarms of robots are expected to feature the following inherent advantages (Şahin et al., 2008; Brambilla et al., 2013):

- **Robustness:** The swarm is robust to the loss or failure of individual robots.
- **Flexibility:** The swarm can reconfigure to tackle different tasks.
- **Scalability:** The swarm can grow and shrink in size depending on the needs of the global task.

When designing a swarm of MAVs, we must then ask ourselves: how can we design a swarm that is robust, flexible, and scalable? It is true that these properties pertain to the swarm rather than the individual, but if the swarm is composed of individual units, then it follows that they must also be present (although perhaps not always apparent) at the local level. We cannot use individual robots that are not robust and merely expect the swarm as a whole to be immune or tolerant to individual failures (Bjerknes and Winfield, 2013). If there is a high probability of errors at the local level, such as erroneous observations, poorly executed commands, or failure of a unit, then this may have a repercussion on the swarm's performance; an effect that Bjerknes and Winfield (2013) have shown can worsen with the number of robots in a swarm. There is a point after which the individual robots are too unreliable and the swarm can fail to achieve its goal, or it can be shown to be outperformed by smaller teams with more reliable units (Stancliff et al., 2006) or even by a single reliable system (Engelen et al., 2014). The further complication with MAVs is that local failures do not remain local, but are likely to cause collisions and damages to other nearby MAVs and/or objects. For some tasks, such as collective transport, the impact may be even more severe as the MAVs are mechanically attached to the load (Tagliabue et al., 2019). It thus follows that, to develop a robust swarm for real world deployment, we must also ensure robustness at the local level.

Of equal importance is to make sure that the robots have the satisfactory tools and sensors to carry out their individual components of a global task. The more capable the sensors are, the more likely it is that the swarm can be flexible and adjust to different tasks or unexpected changes. When performing pure swarm intelligence research, we can afford to abstract away from lower level issues (Brutschy et al., 2015). For instance, in a study on making a decision about selecting a new location for a swarm's nest, one can abstract away from actually evaluating the quality of a nest location, and instead focus the analysis on a particular aspect of the system, such as the decision making process. However, when dealing with real-world applications, this is not an option. If we want to develop nest selection capabilities for a swarm in the real world, each robot should be capable of: flying and operating safely, recognizing the existence of a site, evaluating the quality of a site with a certain reliability, exchanging this information with its neighbors, and more. All these lower level requirements need to be appropriately realized for the global level outcome to emerge, or otherwise need to be accepted as limitations of the system. The way in which they are implemented shape the final behavior of the swarm.

Last but not least, unless properly accounted for, there are scalability problems that may also occur as the swarm grows in size. Examples of issues are: a congested airspace whereby the MAVs are unable to adhere to safety distances, a cluttered visual environment as a result of several MAVs (thus obstructing the task), or poor connectivity as a result of low-range communication capabilities. To achieve scalability, the MAV design must be such that these properties are appropriately accommodated, from the appropriate hardware design all the way to the higher level controllers which make up the swarm behavior.

### 2.1. The Challenge of Local Sensing and Control

When flying several MAVs at once, the control architecture can be of two types: (1) centralized, or (2) decentralized. In the centralized case, all MAVs in a swarm are controlled by a single computer. This “omniscient” entity knows the relevant states of all MAVs and can (pre-)plan their actions accordingly. The planning can be done a-priori and/or online. In the decentralized case, the MAVs make their decisions locally.

A second dichotomy can also be defined for how the MAVs sense their environment: (1) using external position sensing, or (2) locally. External positioning is typically achieved with a Global Navigation Satellite System (GNSS) or with a Motion Capture System (MCS), depending on whether the MAVs are flying outdoors or indoors, respectively. Alternatively, the latter only relies on the sensors that are on-board of the MAV.

Currently, the combination of centralized architecture and external positioning have achieved the highest stage of maturity, allowing for flights with several MAVs. Kushleyev et al. (2013) showed a swarm of 20 micro quadrotors that could re-organize in several formations. Lindsey et al. (2012), Augugliaro et al. (2014), and Mirjan et al. (2016) developed impressive collaborative construction schemes using a team of MAVs. Preiss et al. (2017) showcased “Crazyswarm,” an indoor display of 49 small quadrotors flying together. The strategy of centralized planning and external positioning has also attracted large industry investments, leading to shows with record-breaking number of MAVs flying simultaneously. In 2015, Intel and Ars Electronica Futurelab first flew 100 MAVs, making a Guinness World Record (Swatman, 2016a). In 2016, Intel beat its own record by flying 500 MAVs simultaneously (Swatman, 2016b). In 2018, EHang claimed the record with 1,374 MAVs flying above the city of Xi'an, China (Cadell, 2018). In 2019, Intel reclaimed the title by flying 2,066 MAVs (Guinness World Records, 2018) outdoors. Meanwhile, the record for the most MAVs flying indoors (from a single computer) was recently broken by BT with 160 MAVs (Guinness World Records, 2019).

**Figure 1:**
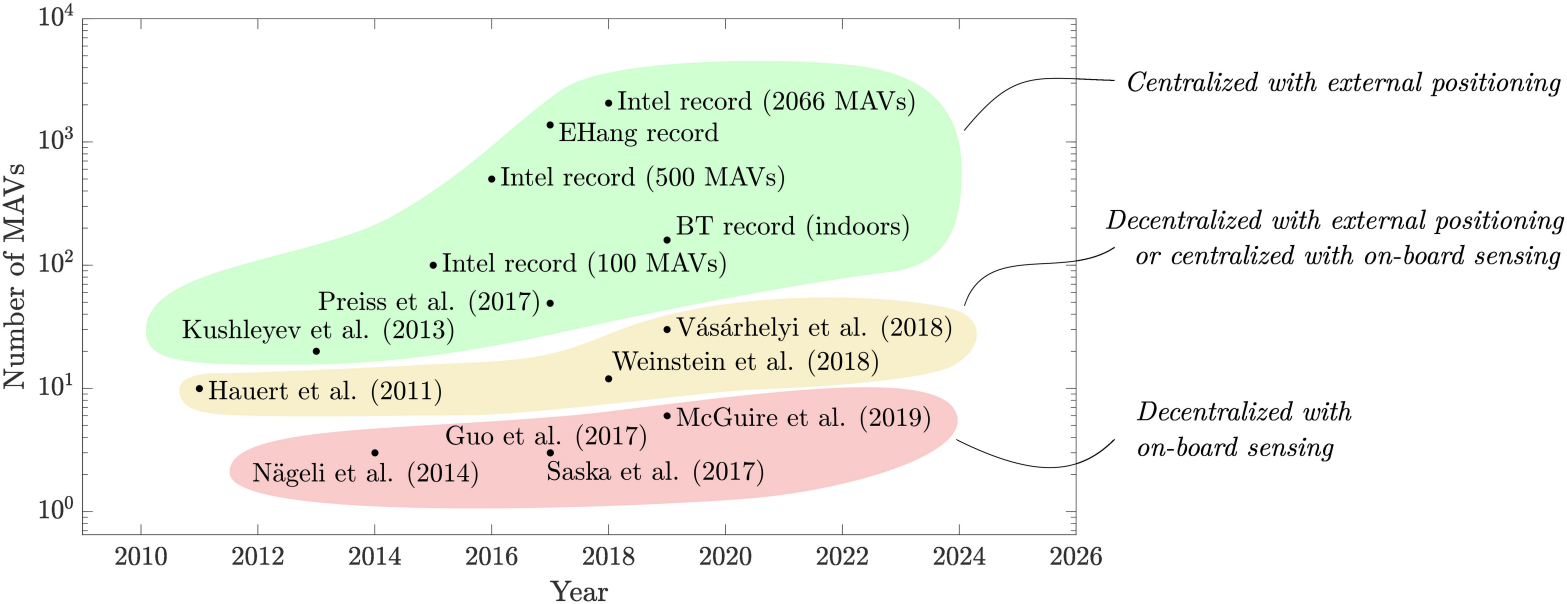
Scatter plot of the number of MAVs that have been flown in sampled state of the art studies discussed in this paper. The combination of centralized planning/control with external positioning has allowed to fly significantly larger swarms. The numbers are lower for the works featuring decentralized control with external positioning, or centralized control with local sensing. The works that use both decentralized control and do not rely on external positioning can be seen to feature the fewest MAVs due to the increased complexity of the control and perception task.

Without external positioning systems or centralized planning/control, the problem of flying several MAVs at once becomes more challenging. This is because: (1) the MAVs have to rely only on on-board perception, or (2) they have to make local decisions without the benefit of global planning, or (3) both. It is then not surprising that, as shown in Figure 1, the swarms that have been flown without external positioning and/or centralized control are significantly smaller. When the control is decentralized, but the MAVs benefit from an external positioning system, or vice versa, the largest swarms are in the dozens (Hauert et al., 2011; Vásárhelyi et al., 2018; Weinstein et al., 2018). For swarms featuring both local perception and distributed control, the highest numbers are currently in the single digits (Nägeli et al., 2014; Guo et al., 2017; Saska et al., 2017; McGuire et al., 2019). Despite the fact that these numbers have been increasing in the last few years, they are still lower, as the operations are shifted away from external system and toward on-board perception and control. If the past is any indication for the future, we expect that: (1) the numbers of drones will keep increasing for all cases, and (2) businesses will take over the records as the technologies for on-board decision making and perception become more mature.

Although we can fly a high number of MAVs when using centralized planning and external positioning, swarming is not just a numbers game. Flying with many MAVs does not automatically imply that we are achieving the benefits of swarm robotics (Hamann, 2018). A centralized system relies on a main computer to take all decisions. This means that a prompt online re-planning is needed in order to achieve robustness and flexibility. This re-planning grows in complexity with the size of the swarm, making the system unscalable. Moreover, the central computer represents a single point of failure. Instead, a swarm adopts a distributed strategy whereby each robot takes a decision independently. The fact that each MAV needs to take its own decisions, and additionally, if the MAVs do not rely on external infrastructure, introduces a new layer of difficulty. However, this is also what brings new advantages: redundancy, scalability, and adaptability to changes (Şahin, 2005; Bonabeau and Théraulaz, 2008)^1^.

When we analyze swarms of MAVs with local on-board sensing and control, we can observe two trends: (1) As the size of the swarm increases, the *relative* knowledge that each MAV will have of its global environment, which includes the remainder of the swarm, decreases (Bouffanais, 2016); (2) as the individual MAV's size and/or mass decreases, its capability to sense its own local environment decreases (Kumar and Michael, 2012). This creates an interesting challenge. On the one hand, we aim to design smaller, lighter, cheaper, and more efficient MAVs. On the other hand, as we make these MAVs smaller, the gap between the microscopic and macroscopic widens further. Designing the swarm becomes a more challenging task because each MAV has less information about its environment and is also less capable to act on it. This can be generalized to other robotic platforms as well, but MAVs feature the increased difficulty of having a tightly bound relationship between their on-board capabilities, their dynamics, their processing power, and their sensing (Chung et al., 2018). This is sometimes referred to as the SWaP (Size, Weight, and Power) trade-off (Mahony et al., 2012; Liu et al., 2018). The relationship is often non-linear. For instance, if we add a sensor that results in 5% more power usage, it does not only spend more energy per second, but it also affects the total energy that can be extracted from the battery as it will be operating in a different regime (de Croon et al., 2016). For many MAVs, grams and milliwatts matter. This makes the design of autonomous decentralized swarms of MAVs a more unique challenge.

### 2.2. Overview of Design Challenges Throughout the Design Chain

Throughout this paper, we shall review the state of the art in MAV technology from the swarm robotics perspective. To facilitate our discussion, we will break down the challenges for the design and control of an MAV swarm in the following four levels, from “local” to “global.”

1. MAV design. This defines the processing power, flight time, dynamics, and capabilities of the single MAV. Most importantly from a swarm engineering perspective, it defines the sensory information available on each unit, from which it can establish its view of the world. This is discussed in section 3.
2. Local ego-state estimation and control. At the lowest level, an MAV must be capable of controlling its motion with sufficient accuracy. This lower level layer handles basic flight operations of the MAV. This includes attitude control, height control, and velocity estimation and control. Moreover, the MAV should be capable of safely navigating in its environment. Minimally, it should detect and avoid potential obstacles. The challenges and state of the art for these methods are discussed in section 4.
3. Intra-swarm relative sensing and avoidance. There are two key enabling technologies for swarming. The first is the knowledge on (the location of) nearby neighbors. This is particularly important for MAV swarms as it not only enables several higher level swarming behaviors, but it also ensures that MAVs do not collide with one another in mid-air. The second enabling technology is communication between MAVs, such that they can share information and thus expand their knowledge of the environment via their neighborhood. These are is discussed in section 5.
4. Swarm behavior. This is the higher level control policy that the robots follow to generate the global swarm behavior. Examples of higher level controllers in swarms range from attraction and repulsion forces for flocking (Gazi and Passino, 2002; Vásárhelyi et al., 2018) to neural networks for aggregation, dispersion, or homing (Duarte et al., 2016). We discuss how MAV swarm behaviors can be designed in section 6.

Other similar taxonomies have been defined. Floreano and Wood (2015) describe three levels of robotic cognition: sensory-motor autonomy, reactive autonomy, and cognitive autonomy. Meanwhile, de Croon et al. (2016) divide the control process for autonomous flight into four levels: attitude control, height control, collision avoidance, and navigation. Although the taxonomies above are conceptually similar (generally going from low level sensing and control to a higher level of cognition), the re-definition that we provide here is designed to better organize our discussion within the context of swarm robotics. Moreover, we also include the design of the MAV within the chain. As we will explain in this manuscript, this has a fundamental impact on the higher level layers.

**Figure 2:**
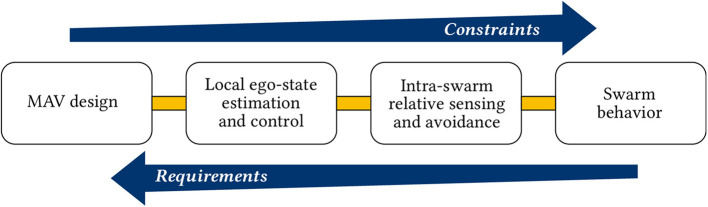
Generalized depiction of the flow of requirements and constraints for the design of MAV swarms. The lower level design choices create constraints on the higher level properties of the swarm. Higher level design choices create requirements for the lower levels, down to the physical design of the MAV. Note that, for the specific case, the flow of requirements and constraints is likely to be more intricate than this picture makes it out to be. However, the general idea remains.

The four stages that have been defined have an increasing level of abstraction. The lower levels enable the robustness, flexibility, and scalability properties expected at the higher level, while the higher levels dictate, accommodate, and make the most out of the capabilities set at the lower level. From a more systems perspective, the MAV design poses constraints on what the higher level controllers can expect to achieve, while the higher level controllers create requirements that the MAV must be able to fulfill. A simplified view of the flow of requirements and constraints is shown in Figure 2.

Throughout the remainder of this paper, as we discuss the state of the art at each level, we will highlight the major constraints that flow upwards and the requirements that flow downwards. Naturally, each sub-topic that we will treat features a plethora of solutions, challenges, and methods, each deserving of a review paper of its own. It is beyond the scope (and probably far beyond any acceptable word limit, too) to present an exhaustive review about each topic. Instead, we keep our focus to highlighting the main methodologies and how they can be used to design swarms of MAVs. Where possible, we will refer the reader to more in-depth reviews on a specific topic.

## 3. MAV Design

The differentiating challenge faced by a flying robot, namely (and somewhat trivially) the fact that it has to carry its own mass around, creates a strong design driver toward minimalism. Despite battery mass consisting of up to 20–30% of the total system mass, the flight time of quadrotor MAVs still remains limited to the order of magnitude of minutes (Kumar and Michael, 2012; Mulgaonkar et al., 2014; Oleynikova et al., 2015). To increase the carrying capabilities of an MAV, enabling it to carry more/better sensors, processors, or actuators, while keeping flight time constant, means that the size of the battery should also increase. In turn, this leads to a new increase in mass, and so on. This type of spiral, often referred to as the “snowball effect,” is a well-known issue for the design of any flying vehicle, from MAVs to trans-Atlantic airliners (Obert, 2009; Lammering et al., 2012; Voskuijl et al., 2018). It then becomes paramount for an MAV design to be as minimalist as possible relative to its task, such that it may fulfill the mission requirements with a minimum mass (or, at the very least, there is a trade-off to be considered). This design driver has been taken to the extreme and has lead to the development of miniature MAV systems, popular examples of which include the Ladybird drone and the Crazyflie (Lehnert and Corke, 2013; Remes et al., 2014; Giernacki et al., 2017). These MAVs have a mass of <50 g, making them attractive due to their low cost and the fact that they are safer to operate around people. This makes them appealing for swarming, especially in indoor environments (Preiss et al., 2017).

A substantial body of literature already exists on single MAV design, the specifics of which largely vary depending on the type of MAV in question. We refer the reader to the works of Mulgaonkar et al. (2014) and Floreano and Wood (2015) and the sources therein for more details. From the swarming perspective, it is important to understand that, independently of the type of MAV in question, the following constraints are intertwined during the design phase: (1) flight time, (2) on-board sensing, (3) on-board processing power, and (4) dynamics. This means that the choice of MAV directly constrains the application as well as the swarming behavior that can be achieved (or, vice versa, a desired swarming behavior requires a specific type of MAV). For example, fixed wing MAVs benefit from longer autonomy. This makes them ideal candidates for long term operations, and also give the operators more time to launch an entire fleet and replace members with low batteries (Chung et al., 2016). However, fixed wing MAVs also have limited agility in comparison to quadrotors or flapping wing MAVs. The latter, for instance, can have a very high agility (Karásek et al., 2018), but also comes with more limited endurance and payload constraints (Olejnik et al., 2019). The MAV design impacts the number and type of sensors that can be taken on-board. It can also impact how these sensors are positioned and their eventual disturbances and noise. In turn, this affects the local sensing and control properties of the MAV and can also impact its ability to sense neighbors and operate in a team more effectively. We will return to this where relevant in the next chapters, whereby we discuss how an MAV can estimate and control its motion, sense its neighbors, and navigate in an environment together with the rest of the swarm.

A special note is made to designs that are *intended* for collaboration. Oung and D'Andrea (2011) introduced the Distributed Flight Array, a design whereby multiple single rotors can attach and detach from each other to form larger multi-rotors. More recently, Saldaña et al. (2018) introduced the ModQuad: a quadrotor with a magnetic frame designed for self-assembly with its neighbors. This design provides a solution for collaborative transport by creating a more powerful rigid structure with several drones. Gabrich et al. (2018) have shown how the ModQuad design can be used to form an aerial gripper. Because of the frame design, one of the difficulties of the ModQuad was in the disassembly back to individual quadrotors. This was tackled with a new frame design which enabled the quadrotors to disassemble by moving away from each other with a sufficiently high roll/pitch angle (Saldaña et al., 2019).

## 4. Local Ego-State Estimation and Control

The primary objective for a single MAV operating in a swarm is to remain in flight and perform higher level tasks with a given accuracy. This requires a robust estimation of the on-board state as well as robust lower level control, preferably while minimizing the size, power, and processing required. The design choices made here dictate the accuracy (i.e., noise, bias, and disturbances) with which each MAV will know its own state, as well as which variables the state is actually comprised of. In turn, this affects the type of maneuvers and actions that an MAV can execute. For instance, aggressive flight maneuvers likely require relatively accurate real-time state estimation (Bry et al., 2015). Of equal importance are the considerations for the processing power that remains for higher level tasks. While it can be attractive to implement increasingly advanced algorithms to achieve a more reliable ego-state estimate, these can be too computationally expensive to run on-board even by modern standards (Ghadiok et al., 2012; Schauwecker and Zell, 2014). This limits the MAV, as processing power is diverted from tasks at a higher level of cognition. If not properly handled, it can lead to sub-optimal final performances by the MAVs and by the swarm^2^.

### 4.1. Low-Level State Estimation and Control

This section outlines the main sensors and methods that can be used by MAVs to measure their on-board states, laying the foundations for our swarm-focused discussion in later sections. We organize the discussion by focusing on the following parameters: attitude (section 4.1.1), velocity and odometry (section 4.1.2), and height and altitude (section 4.1.3). Moreover, we restrict our overview to *on-board* sensing, as this is in line with the swarming philosophy and the relevant applications.

#### 4.1.1. Attitude

It is essential for an MAV to estimate and control its own attitude in order to control its flight (Beard, 2007; Bouabdallah and Siegwart, 2007). Accelerations and angular rotation rates are typically measured through the on-board Inertial Measurement Unit (IMU) sensor (Bouabdallah et al., 2004; Gupte et al., 2012). The IMU measurements can be fused together to both estimate and control the attitude of an MAV (Shen et al., 2011; Schauwecker et al., 2012; Macdonald et al., 2014; Mulgaonkar et al., 2015). Additionally to the IMU, MAVs equipped with cameras can also use it to infer the attitude with respect to certain reference features or planar surfaces, as in Schauwecker and Zell (2014). Thurrowgood et al. (2009), Dusha et al. (2011), de Croon et al. (2012), and Carrio et al. (2018) estimate the roll and pitch angles of an MAV based on the horizon line (outdoors). The measurements from the IMU and vision can then be filtered together to improve the estimate as well as filter out the accumulating bias from the IMU (Martinelli, 2011). Once known, attitude control can be achieved with a variety of controllers. For a recent survey that treats the topic of attitude control in more detail, we refer the reader to the review by Nascimento and Saska (2019). Of particular interest to swarming are controllers that can provide robustness to disturbances or mishaps. One interesting example is the scheme devised by Faessler et al. (2015), which can automatically re-initialize the leveled flight of an MAV in mid-air.

Measuring and controlling the heading (for instance, with respect to North) is not strictly needed for basic flight. However, it can be an enabler for collective motion by providing a common reference that can be measured locally by all MAVs (Flocchini et al., 2008). Heading with respect to North can be measured with a magnetometer, which is a common component for MAVs (Beard, 2007). A main limitation of this sensor is that it is highly sensitive to disturbances in the environment (Afzal et al., 2011). The disturbances can be corrected for with the use of other attitude sensors. For example, Pascoal et al. (2000) fused gyroscope measurements with the magnetometer in order to filter out disturbances from the magnetometer while also reducing the noise from the gyroscope. Another sensor that has been explored is the celestial compass, which extracts the orientation based on the Sun (Jung et al., 2013; Dupeyroux et al., 2019). Although this sensor is not subject to electro-magnetic disturbances, it is limited to outdoor scenarios and performs best under a clear sky, which may also not always be the case.

#### 4.1.2. Velocity and Odometry

A tuned sensor fusion filter with an accurate prediction model can estimate velocity just based on the IMU readings (Leishman et al., 2014). However, the use of additional and dedicated velocity sensors is commonly used to achieve a more robust system without bias. Fixed wing MAVs can be equipped with a pitot tube in order to measure airspeed (Chung et al., 2016). For other designs, such as quadrotors, a popular solution is to measure the optic flow, i.e., the motion of features in the environment, from which an MAV can extract its own velocity (Santamaria-Navarro et al., 2015). To observe velocity, the flow needs to be scaled with the help of a distance measurement, such as height (albeit this assumes that the ground is flat, which may be untrue in cluttered/outdoor environments). Optic flow can be measured with a camera or with dedicated sensors, such as PX4FLOW (Honegger et al., 2013) or the PixArt sensor^3^. Using optical mouse sensors, Briod et al. (2013) were able to make a 46 g quadrotor fly based on only inertial and optical-flow sensors, even without the need to scale the flow by a distance measurement. This was achieved by only using the direction of the optic flow and disregarding its magnitude. In nature, optic flow has also been shown to be directly correlated with how insects control their velocity in an environment (Portelli et al., 2011; Lecoeur et al., 2019). Similar ideas have also been ported to the drone world, whereby the optic flow detection is directly correlated to a control input, without even necessarily extracting states from it (Zufferey et al., 2010). This can be an attractive property in order to create a natural correlation between a sensor and its control properties. State estimates improve when optic flow is fused with other sensors, such as IMU readings or pressure sensors (Kendoul et al., 2009a,b; Santamaria-Navarro et al., 2015), or with the control input of the drone (Ho et al., 2017). As opposed to optic flow sensors, a camera has the advantage that it can observe both optic flow as well as other features in the environment, thus enabling an MAV to get more out of a single sensor. Although this is more computationally expensive, it also provides versatility.

The use of vision also enables the tracking of features in the environment, which a robot can use to estimate its odometry. Using Visual Odometry (VO), a robot integrates vision-based measurements during flight in order to estimate its motion. The inertial variant of VO, known as Visual Inertial Odometry (VIO), further fuses visual tracking together with IMU measurements. This makes it possible for an MAV to move accurately relative to an initial position (Scaramuzza and Zhang, 2019). VIO has been exploited for swarm-like behaviors, such as in the work by Weinstein et al. (2018), whereby twelve MAVs form patterns by flying pre-planned trajectories and use VIO to track their motion. A step beyond VO and its variants is to use Simultaneous Localization And Mapping (SLAM). The advantage of SLAM is that it can mitigate the integration drift of VO-based methods. When solving the full SLAM problem, a robot estimates its odometry in the environment and then corrects it by recognizing previously visited places and optimizing the result accordingly, so as to make a consistent map (Cadena et al., 2016; Cieslewski and Scaramuzza, 2017). Yousif et al. (2015) and Cadena et al. (2016) provide more in-depth reviews of VO and SLAM algorithms. Within the swarming context, a map can also be shared so as to make use of places and features that have been seen by other members of the swarm. One common drawback of VO and SLAM methods is that they are computationally intensive and thus reserved for larger MAVs (Ghadiok et al., 2012; Schauwecker and Zell, 2014). However, recent developments have also seen the introduction of more light-weight solutions, such as Navion (Suleiman et al., 2018).

Odometry and SLAM are not limited to the use of vision. A viable alternative sensor is the LIDAR (Light Detection and Ranging) scanner, more commonly referred to as “laser scanner.” LIDAR-based SLAM feature the same philosophy as the vision counterparts, but instead of a camera it uses LIDAR to measure depth information and build a map (Bachrach et al., 2011; Opromolla et al., 2016; Doer et al., 2017; Tripicchio et al., 2018). A LIDAR is generally less dependent on lighting conditions and needs less computations, but it is also heavier, more expensive, and consumes more on-board power (Opromolla et al., 2016). Vision and LIDAR can also be used together to further enhance the final estimates (López et al., 2016; Shi et al., 2016).

#### 4.1.3. Height and Altitude

In an abstract sense, the ground represents an obstacle that the MAV must avoid, much like walls, objects, or other MAVs. It does not need to be explicitly known in order to control an MAV, as shown in the work of Beyeler et al. (2009). Unlike other obstacles, however, gravity continuously pulls the MAV toward the ground, meaning that measuring and controlling height and altitude often requires special attention.

Note that we differentiate here between height and altitude. Height is the distance to the ground surface, which can vary when there is a high building, a canyon, or a table. The height of an MAV can be measured with an ultrasonic range finder (or “sonar”). Sonar can provide more accurate data at the cost of power, mass, size, and a limited range. Its accuracy, however, made it a part of several designs (Krajník et al., 2011; Ghadiok et al., 2012; Abeywardena et al., 2013). Infra-red or laser range finders have also been used as an alternative (Grzonka et al., 2009; Gupte et al., 2012). The advantage of an infra-red sensor is that it can be very power efficient, albeit it is only reliable up to a limited range of a few meters, and on favorable light conditions (Laković et al., 2019)^4^. Altitude is the distance to a fixed reference point, such as sea level or a take-off position. A pressure sensor is a common sensor to obtain this measurement (Beard, 2007), but it can be subject to large noise and disturbances in the short term, which can be reduced via low pass filters (Sabatini and Genovese, 2013; Shilov, 2014). If flying outdoors, a Global Navigation Satellite System (GNSS) can also be used to obtain altitude.

The choice of height/altitude sensor has an impact on the swarm behaviors that can be programmed. GNSS and pressure sensors provide a measurement of the altitude of the MAV with respect to a certain position. This is an attractive property, although, as previously discussed, GNSS is limited to outdoor environments, while pressure sensors can be noisy. Moreover, all pressure sensors of all MAVs in the swarm should be equally calibrated. Unlike pressure sensors, ultrasonic sensors or laser range finders do not require this calibration step, since the measurement is made from the MAV to the nearest surface. However, one must then assume that the MAVs all fly on a flat plane with no objects (or other MAVs below them), which may turn out to not be a valid assumption. SLAM and VIO methods, previously discussed in section 4.1.2, can also estimate altitude/height as part of the odometry/mapping procedure provided that a downwards facing camera is available.

Just as for the use of a common heading like North, the measurements of height and/or altitude can provide a common reference plane for a swarm of MAVs. If the vertical distance between the MAVs is sufficient, it can provide a relatively simple solution for intra-swarm collision avoidance (albeit with constraints—we return to this in section 5.2). It can also enable self-organized behaviors, such as in the work of Chung et al. (2016), where the MAVs are made to follow the one with the highest altitude within their sub-swarm. In this way, the leader is automatically elected in a self-organized manner by the swarm. For example, should a current leader MAV need to land as a result of a malfunction, a new leader can be automatically re-elected so that the rest of the swarm can keep operating.

### 4.2. Achieving Safe Navigation

It is important that each MAV remains safe and that it does not collide with its surroundings, or that damages remain limited in case this happens. This safety requirement can be satisfied in two ways. The first, which is more “passive” and brings us back to MAV design, is to develop MAVs that are mechanically collision resilient. This allows the MAV to hit obstacles without risking significant damage to itself or its environment. With this rationale, Briod et al. (2012), Mulgaonkar et al. (2015, 2018), and Kornatowski et al. (2017) placed protective cages around an MAV. However, the additional mass of a cage can negatively impact flight time and the cage can also introduce drag and controllability issues (Floreano et al., 2017). Instead, Mintchev et al. (2017) developed a flexible design for miniature quadrotors in order to be more collision resilient upon impact with walls. The use of airships has also been proposed as a more collision resilient solution (Melhuish and Welsby, 2002; Troub et al., 2017). The limitations of airships, however, are in their lower agility and restricted payload capacity. More recently, Chen et al. (2019) demonstrated insect scale designs that use soft artificial muscles for flapping flight. The soft actuators, combined with the small scale of the MAV, are such that the MAVs can be physically robust to collisions with obstacles and with each other. Collision resistant designs can even be exploited to improve on-board state estimation, such as in the recent work by Lew et al. (2019), whereby collisions are used as pseudo velocity measurement under the assumption that the velocity perpendicular to an obstacle, at the time of impact, is null. The alternative, or complementary, solution to passive collision resistance is “active” obstacle sensing and avoidance, whereby an MAV uses its on-board sensors to identify and avoid obstacles in the environment.

Collision-free flight can be achieved via two main navigation philosophies: (1) map-based navigation, and (2) reactive navigation. With the former, a map of the environment can be used to create a collision-free trajectory (Shen et al., 2011; Weiss et al., 2011; Ghadiok et al., 2012). The map can be generated during flight (using SLAM) and/or, for known environments, it can be provided a-priori. The advantage of a map-based approach is that obstacle avoidance can be directly integrated with higher level swarming behaviors (Saska et al., 2016b). Instead, a reactive control strategy uses a different philosophy whereby the MAV only reacts to obstacles in real-time as they are measured, regardless of its absolute position within the environment. In this case, if an MAV detects an obstacle, it reacts with an avoidance maneuver without taking its higher level goal into account. The trajectories pursued with a reactive controller may be less optimal, but the advantage of a reactive control strategy is that it naturally accounts for dynamic obstacles and it is not limited to a static map. The two can also operate in a hierarchical manner, such that the reactive controller takes over if there is a need to avoid an obstacle, and the MAV is otherwise controlled at a higher level by a path planning behavior. Regardless of the navigation philosophy in use, if the MAV needs to sense and avoid obstacles during flight, it will require sensors that can provide it with the right information in a timely manner.

Of all sensors, vision provides a vast amount of information from which an MAV can interpret its direct environment. By using a stereo-camera, the disparity between two images gives depth information (Heng et al., 2011; Matthies et al., 2014; Oleynikova et al., 2015; McGuire et al., 2017). Alternatively, a single camera can also be used. For example, the work of de Croon et al. (2012) exploited the decrease in the variance of features when approaching obstacles. Ross et al. (2013) used a learning routine to map monocular camera images to a pilot command in order to teach obstacle avoidance by imitating a human pilot. Kong et al. (2014) proposed edge detection to detect the boundary of potential obstacles in an image. Saha et al. (2014) and Aguilar et al. (2017) used feature detection techniques in order to extract potential obstacles from images. Alvarez et al. (2016) used consecutive images to extract a depth map (a technique known as “motion parallax”), albeit the accuracy of this method is dependent on the ego-motion estimation of the quadrotor. Learning approaches have also been investigated in order to overcome the limitations of monocular vision. By exploiting the collision resistant design of a Parrot AR Drone, Gandhi et al. (2017) collected data from 11,500 crashes and used a self-supervised learning approach to teach the drone how to avoid obstacles from only a monocular camera. Self-supervised learning of distance from monocular images can also be accomplished without the need to crash, but with the aid of an additional sensor. Lamers et al. (2016) did this by exploiting an infrared range sensor, and van Hecke et al. (2018) applied this to see distances with one single camera by learning a behavior that used a stereo-camera. This is useful if the stereo-camera were to malfunction and suddenly become monocular. Alternative camera technologies have also been developed, providing new possibilities. RGB-D sensors are cameras that also provide a per-pixel depth map, a mainstream example of which is the Microsoft Kinect camera (Newcombe et al., 2011). This particular sensor augments one RGB camera with an IR camera and an IR projector, which together are capable of measuring depth (Smisek et al., 2013). RGB-D sensors have been used on MAVs to navigate in an environment and avoid obstacles (Shen et al., 2014; Stegagno et al., 2014; Odelga et al., 2016; Huang et al., 2017). One of the disadvantages of these RGB-D sensors over a stereo-camera set-up (whereby depth is inferred from the disparity) is that RGB-D sensors can be more sensitive to natural light, and may thus perform less well in outdoor environments (Stegagno et al., 2014). Finally, in recent years, the introduction of Dynamic Vision Sensor (DVS) cameras has also enabled new possibilities for reactive obstacle sensing. A DVS camera only measures changes in the brightness, and can thus provide a higher data throughput. This enables a robot to quickly react to sudden changes in the environment, such as the appearance of a fast moving obstacle (Mueggler et al., 2015; Falanga et al., 2019a).

The capabilities of a vision algorithm will depend on the resolution of the on-board cameras, the number of the on-board cameras, as well as the processing power on-board. On very lightweight MAVs, such as flapping wings, even carrying a small stereo-camera can be challenging (Olejnik et al., 2019). A further known disadvantage of vision is the limited Field of View (FOV) of cameras. Omni-directional sensing can only be achieved with multiple sets of cameras (Floreano et al., 2013; Moore et al., 2014) at the cost of additional mass, the impact of which is dependent on the design of the MAV.

Although vision is a rich sensor, in that it can provide different types of information, other sensors also can be used for reactive collision avoidance. LIDAR, for instance, has the advantage that it is less dependent on lighting conditions and can provide more accurate data for localization and navigation (Bachrach et al., 2011; Tripicchio et al., 2018). Alternatively, time-of-flight laser ranging sensors have also been proposed for reactive obstacle avoidance algorithms on small drones (Laković et al., 2019). These uni-directional sensors can sense whether an object appears along their line of sight (typically up to a few meters). Due to their small size and low power requirements, they can be used on tiny MAVs (Bitcraze, 2019)^5^.

## 5. Intra-Swarm Relative Sensing and Collision Avoidance

Once we have an MAV design that can perform basic safe flight, we begin to expand its capabilities toward collaboration in a swarm. Two fundamental challenges need to be considered in this domain. The first is relative localization. This is not only required to ensure intra-swarm collision avoidance, which is a basic safety requirement, but also to enable several swarm behaviors (Bouffanais, 2016). The design choice used for intra-swarm relative localization defines and constrains the motion of the MAVs relative to one another, which affects the swarming behavior that can be implemented. The second challenge is intra-swarm communication. Much like knowing the position of neighbors, the exchange of information between MAVs can help the swarm to coordinate (Valentini, 2017; Hamann, 2018). In this section, we explore the state of the art for relative localization (section 5.1), reactive collision avoidance maneuvers (section 5.2), and we discuss intra-swarm communication technologies (section 5.3).

### 5.1. Relative Localization

In outdoor environments, relative position can be obtained via a combination of GNSS and intra-swarm communication. Global position information obtained via GNSS is communicated between MAVs and then used to extract relative position information. This has enabled connected swarms that can operate in formations or flocks (Chung et al., 2016; Yuan et al., 2017). An impressive recent display of this in the real world was put into practice by Vásárhelyi et al. (2018), who programmed a swarm of 30 MAVs to flock. The same concept can be applied to indoor environments if pre-fitted with, for example: external markers (Pestana et al., 2014), motion-tracking cameras (Kushleyev et al., 2013), antenna beacons (Ledergerber et al., 2015; Guo et al., 2016), or ultra sound beacons (Vedder et al., 2015). However, this dependency on external infrastructure limits the swarm to being operable only in areas that have been properly fitted to the task. Several tasks, especially the ones that involve exploration, cannot rely on these methods. In order to remove the dependency on external infrastructure, there is a need for technologies that allows the MAVs themselves to obtain a direct MAV-to-MAV relative location estimate. This is still an open challenge, with several technologies and sensors currently being developed.

One of the earlier solutions for direct relative localization on flying robots proposed the use of infrared sensors (Roberts et al., 2012). However, since infrared sensors are uni-directional, this used an array of sensors (both emitting and receiving) placed around the MAV in order to approach omni-directionality, making for a relatively heavy system. Alternatively, vision-based algorithms have once again been extensively explored. However, the robust visual detection of neighboring MAVs is not a simple task. The object needs to be recognized at different angles, positions, speeds, and sizes. Moreover, the image can be subject to blur or poor lighting conditions. One way to address this challenge is with the use of visual aids mounted on the MAVs, such as visual markers (Faigl et al., 2013; Krajník et al., 2014; Nägeli et al., 2014), colored balls (Roelofsen et al., 2015; Epstein and Feldman, 2018), or active markers, such as infrared markers (Faessler et al., 2014; Teixeira et al., 2018)^6^ or Ultra Violet (UV) markers (Walter et al., 2018, 2019). Visual aids simplify the task and improve the detection accuracy and reliability. However, they are not as easily feasible on all designs, such as flapping wing MAVs or smaller quadrotors. Marker-less detection of other MAVs is very challenging, since other MAVs have to be detected against cluttered, possibly dynamic backgrounds while the detecting MAV is moving by itself as well. A successful current approach is to rely on stereo vision, where other drones can be detected because they “float” in the air unlike other objects like trees or buildings. Carrio et al. (2018) explored a deep learning algorithm for the detection of other MAVs in stereo-based disparity images. An alternative is to detect other MAVs in monocular still images. Like the detection in stereo disparity images, this removes the difficulty of interpreting complex motion fields between frames, but it introduces the difficulty of detecting other, potentially (seemingly) small MAVs against background clutter. To solve the challenge, Opromolla et al. (2019) used a machine learning framework that exploited the knowledge that the MAVs were supposed to fly in formation. Their scheme used the knowledge of the formation in order to predict the expected position of a neighboring MAV and focus the vision-based detection on the expected region, thus simplifying the task. Employing a more end-to-end learning technique, Schilling et al. (2019) used imitation learning to autonomously learn a flocking behavior from camera images. Following the attribution method by Selvaraju et al. (2017), Schilling et al. studied the influence that each pixel of an input image had on the predicted velocity. It was shown that the parts of the image whereby neighboring MAVs could be seen were more influential, demonstrating that the network had implicitly learned to localize its neighbors. Despite the promising preliminary results, it is yet to be seen how it can handle other MAVs sizes or more cluttered backgrounds. Finally, it is possible to use the optic flow field for detecting other MAVs. This approach could have the benefit of generality, but it would require the calculation and interpretation of a complex, dense optic flow field. To our knowledge, this method has not yet been investigated.

From a swarming perspective, it may also be desirable to know the ID of a neighbor. However, IDs may be difficult to detect using vision without the aid of markers. This issue was explored by Stegagno et al. (2011), Cognetti et al. (2012), and Franchi et al. (2013) with fusion filters that infer IDs over time with the aid of communication. Moreover, cameras have a limited FOV. This limits the behaviors that can be achieved by the swarm. For instance, it may be limiting for surveillance tasks where quadrotors may need to look away from each other but can't or else they may collide or disperse. It can be addressed by placing several cameras around the MAVs (Schilling et al., 2019), but at the cost of additional mass, size, and power, which in turn creates new repercussions.

The use of vision is not only limited to directly recognizing other drones in the environment. With the aid of communication, two or more MAVs can also estimate their relative location indirectly by matching mutually observed features in the environment. The MAVs can compare their respective views and infer their relative location. In the most complete case, each MAV uses a SLAM algorithm to construct a map of its environment, which is then compared in full (as discussed in section 4, this can also be accomplished using other sensors, such as LIDAR, so this approach is not only reserved for vision). Although SLAM is a computationally expensive task, more easily handled centrally (Achtelik et al., 2012; Forster et al., 2013), it can also be run in a distributed manner, making for an infrastructure free system (Cunningham et al., 2013; Cieslewski et al., 2018; Lajoie et al., 2019). For a survey of collaborative visual SLAM, we refer the reader to the paper by Zou et al. (2019) and the sources therein. An additional benefit of collective map generation is that the MAVs benefit from the observations of their team-mates and can thus achieve a better collective map. However, if the desired objective is only to achieve relative localization, the computations can be simplified. Instead of computing and matching an entire map, the MAVs need only to concern themselves with the comparison of mutually observed features in order to extract their relative geometric pose (Achtelik et al., 2011; Montijano et al., 2016). This requires that the images compared by the MAVs have sufficient overlap and can be uniquely identified.

An alternative stream of research leverages only communication between MAVs to achieve relative localization, while also using the antennas as relative range sensors. Here, we will refer to these methods as *communication-based ranging*. The advantage of this method is that it offers omni-directional information at a relatively low mass, power, and processing penalty, leveraging a technology that is likely available on even the smallest of MAVs. Szabo (2015) first proposed the use of signal strength to detect the presence of nearby MAVs and engage in avoidance maneuvers. Also for the purposes of collision avoidance, Coppola et al. (2018) implemented a beacon-less relative localization approach based on the signal strength between antennas, using the Bluetooth Low Energy connectivity already available on even the smaller drones. Guo et al. (2017) proposed a similar solution using UltraWide Band (UWB) antennas for relative ranging, which offer a higher resolution even at larger distances. However, this work used one of the drones as a reference beacon for the others. One commonality between the solutions by Guo et al. (2017) and Coppola et al. (2018) was that the MAVs were required to have a knowledge of North, which enabled them to compare each other's velocities along the same global axis. However, in practice this is a significant limitation due to the difficulties of reliably measuring North, especially if indoors, as already discussed in section 4.1.1. To tackle this, van der Helm et al. (2019) showed that, if using a high accuracy ranging antenna, such as UWB, then it is not necessary for the MAVs to measure a common North. However, selecting this option creates fundamental constraints on the high-level behaviors of the swarm. This issue is there for the case where North is known and when it is not, albeit the requirement when North is not known are more stringent. If North is known, at least one of the MAVs must be moving relative to the other for the relative localization to remain theoretically observable. If North is not known, all MAVs must be moving. The MAVs remain bound to trajectories that excite the filter (van der Helm et al., 2019). For the case where North is known, Nguyen et al. (2019) proposed that a portion of MAVs in the swarm should act as “observers” and perform trajectories that persistently excite the system.

Another solution is to use sound. Early research in this domain was performed by Tijs et al. (2010), who used a microphone to hear nearby MAVs. This was explored in more depth by Basiri (2015) using full microphone arrays for relative localization. A primary issue encountered was that the sound emitted by the listening quadrotor would mask the sound of the neighboring MAVs, which were also similar. This was addressed with the use of a “chirp” sound, which can then be easily heard by neighbors, in order to overcome this issue (Basiri et al., 2014, 2016). In recent work, Cabrera-Ponce et al. (2019) proposed the use of a Convolutional Neural Network to detect the presence of nearby MAVs. This is done using a large scale microphone array (Ruiz-Espitia et al., 2018) featuring eight microphones based on the ManyEars framework (Grondin et al., 2013). Specific to sound sensors, the accuracy of the detection depends on how similar the sounds of other MAVs are. Moreover, the localization accuracy depends on the microphone setup. Most works use a microphone array, where the localization accuracy depends on the length of the baseline between microphones, which is inherently limited on small MAVs.

As it can be seen, several different techniques exist. Minimally, these technologies should enable neighboring MAVs to avoid collisions with one another. However, the particular choice of relative localization technology creates a fundamental constraint on the swarm behavior that can be achieved. For example, communication-based ranging methods have unobservable conditions depending on the MAVs' motion, and sound-based localization with microphone arrays will be less accurate when used on smaller MAVs. Similarly, certain swarm behaviors (e.g., one that requires known IDs, or long range distances) may place certain requirements on which technology is best to be used. In Table 1, we outline the major relative localization approaches with their advantages and disadvantages.

**Table 1:** Current technologies in the state of the art for relative localization between MAVs, with their main advantages and disadvantages.

**Technology**	**Sample references**	**Advantages**	**Disadvantages**
Vision (direct, passive)	Faigl et al. (2013) Krajník et al. (2014) Nägeli et al. (2014) Roelofsen et al. (2015) Carrio et al. (2018)	• Rich information • Passive sensor • Possible to extract ID (if using markers or having otherwise visually distinct MAVs) • Lightweight (depending on model) • Scalable (provided environment is not cluttered)	• Computationally expensive • Limited FOV • Dependent on lighting conditions • Dependent on visual clutter in environment • No IDs (if marker-less) • Needs visual line of sight
Observation matching	Achtelik et al. (2011) Montijano et al. (2016)	• No direct line of sight is needed • Thrives in visually cluttered environments • Includes IDs (via communication)	• Active sensor, requires communication • Requires sufficient visual overlap • Computationally expensive • Dependent on lighting conditions
Communication-based ranging	Szabo (2015) Guo et al. (2017) Coppola et al. (2018) van der Helm et al. (2019) Nguyen et al. (2019)	• Low mass • Omni-directional • Possible to extract IDs • Can work in visually cluttered environments • Enables communication of additional data	• Active sensor, requires communication • Needs relative excitation maneuvers • Noisy (depending on sensor) • Difficult to scale due to communication interference
Sound	Basiri et al. (2014, 2016) Cabrera-Ponce et al. (2019)	• Scalable (provided that the sound environment is, or does not become, not cluttered) • Passive sensor • Omni-directional • Can work in visually cluttered environments	• Self-propeller noise • Limited range • Noise pollution (if using “chirps”) • No IDs (if chirp-less) • Limited angular accuracy due to limited baseline between microphones in the array
Infra-red (sensor array)	Roberts et al. (2012)	• Accurate • Computationally simple • Lower dependence on lighting conditions^a^ • Can work in visually cluttered environments	• Heavy • Needs visual line of sight • Many active sensors result in high energy expense
Vision (direct, with active markers)	Faessler et al. (2014) Teixeira et al. (2018) Walter et al. (2018, 2019)	• Accurate • Possible to extract IDs • Lower dependence on lighting conditions • Can work in visually cluttered environments	• Needs visual line of sight • Many active sensors result in high energy expense

a *Roberts et al. (2012) tested the sensor for 0, 500, and 10,000 lux and found <1% relative error between these lighting conditions. However, the sensor was not tested outdoors*.

### 5.2. Intra-Swarm Collision Avoidance

Collision detection and avoidance of objects in the environment has already been discussed in section 4.2. As MAVs operate in teams, relative intra-swarm collision avoidance also becomes a safety-critical behavior that should be implemented. The complexity of this task is that it requires a collaborative maneuver between two or more MAVs.

MAVs operate in 3D space, and thus relative collision avoidance could be tackled by vertical separation. However, particularly in indoor environments where vertical space is limited, vertical avoidance maneuvers may cause undesirable aerodynamic interactions with other MAVs as well as other parts of the environment. For quadrotors, while aerodynamic influence is negligible when flying side-by-side, flying above another will create a disturbance for the lower one (Michael et al., 2010; Powers et al., 2013). Furthermore, emergency vertical maneuvers could also cause a quadrotor to fly too close to the ground, which creates a ground effect and pushes it upwards, or, if indoors, to fly too close to the ceiling, which creates a pulling effect toward the ceiling (Powers et al., 2013). Vertical avoidance may also corrupt the sensor readings of the MAV. For instance, height may be compromised if another MAV obstructs a sonar sensors. Overall, horizontal avoidance maneuvers are desired.

A popular algorithm for obstacle avoidance, provided that the robots know their relative position and velocity, is the Velocity Obstacle (VO) method (Fiorini and Shiller, 1998). The core idea is for a robot to determine a set of all velocities that will lead to collisions with the obstacle (a collision cone), and then choose a velocity outside of that set, usually the one that requires minimum change from the current velocity. VO has stemmed a number of variants specifically designed to deal with multi-agent avoidance, such as Reciprocal Velocity Obstacle (RVO) (van den Berg et al., 2008; van den Berg et al., 2011), Hybrid Reciprocal Velocity Obstacle (HRVO) (Snape et al., 2009), and Optimal Reciprocal Collision Avoidance (ORCA) (Snape et al., 2011). These variants alter the set of forbidden velocities in order to address reciprocity, which may otherwise lead to oscillations in the behavior. These methods have been successfully applied on MAVs, both in a decentralized way as well as via centralized re-planners. They accounted for uncertainties by artificially increasing the perceived radii of the robots. Alonso-Mora et al. (2015) showed the successful use of RVO on a team of MAVs such that they may adjust their trajectory with respect to a reference. This was done using an external MCS for (relative) positioning. Coppola et al. (2018) showed a collision cone scheme with on-board relative localization, introducing a method to adjust the cone angle in order to better account for uncertainties in the relative localization estimates. A disadvantage of VO methods and its derivatives is scalability. If the flying area is limited and the airspace becomes too crowded, then it may become difficult for MAVs to find safe directions to fly toward (Coppola et al., 2018). Another avoidance algorithm, called Human-Like (HL), presents the advantage that the heading selection is decoupled from speed selection (Guzzi et al., 2013a; Guzzi et al., 2014), such that the MAVs only engage in a change in heading. HL has been found to be successful even when operating at relatively lower rates (Guzzi et al., 2013b). Although it has not been tested on MAVs, their tests also demonstrated generally better scalability properties.

Alternatively, attraction and repulsion forces between obstacles are also a valid algorithm for collision avoidance. This is a common technique which has been extensively studied in swarm research (Reynolds, 1987; Gazi and Passino, 2002; Gazi and Passino, 2004). If one wishes for the MAVs to flock, these attraction and repulsion forces can also be directly merged with the swarm controller (Vásárhelyi et al., 2018). One potential short-coming of this approach is that it can lead to equilibrium states whereby the swarm remains in a fixed final formation, although this can also be seen as a positive property that can be exploited (Gazi and Passino, 2011).

In summary, multiple methods exist for intra-swarm collision avoidance. Given sufficiently accurate relative locations, these methods are very successful. The main challenges here are: (1) how to deal with uncertainties and unobservable conditions deriving from the localization mechanism used by the drones, and (2) how to keep guaranteeing successful collision avoidance when the swarm scales up to very large numbers.

### 5.3. Intra-Swarm Communication

Direct sharing of information between neighboring robots is an enabler for swarm behaviors as well as relative sensing (Valentini, 2017; Hamann, 2018; Pitonakova et al., 2018). To achieve the desired effect, it needs to be implemented with scalability, robustness, and flexibility in mind. Common problems that can otherwise arise are: (1) the messaging rate between robots is too low (low scalability); (2) high packet loss (low robustness); (3) communication range is too low (low scalability and flexibility); (4) inability to adapt to a switching network topology (low flexibility) (Chamanbaz et al., 2017).

Solutions to the above depend on the application. With respect to hardware, the three main technologies in the state of the art are: Bluetooth, WiFi, and ZigBee (Bensky, 2019). All three operate in the 2.4*GHz* band^7^. Bluetooth is energy efficient, but features a low maximum communication distances of ≈10–20 m (indoors, depending on the environment and version). This makes it more important to establish a network that can adapt to a switching topology, as it is very likely to change during operations. The latest version of the Bluetooth standard, Bluetooth 5, features a higher range and a higher data-rate despite keeping a low power consumption. It also has longer advertising messages, such that, without pairing, asynchronous network nodes can exchange messages of 255 bytes instead of 31 (Collotta et al., 2018). Bluetooth antennas were used in the previously discussed work of (Coppola et al., 2018) on a swarm of 3 MAVs to exchange data indoors and to measure their relative range. In comparison to Bluetooth, WiFi is known to be less energy efficient, but works more reliably at longer ranges and has a higher data throughput. Chung et al. (2016) used WiFi to enable a swarm of 50 MAVs to form an *ad-hoc* network. WiFi was also used by Vásárhelyi et al. (2018) in combination with an XBee module^8^ using a proprietary communication protocol. ZigBee's primary benefits are scalability (it can keep up to, theoretically, 64,000 nodes) and low power, although it has a low data communication rate (Bensky, 2019)^9^. Depending on the application, this may or may not be an issue depending on what the intra-swarm communication requirements are. Allred et al. (2007) used a ZigBee module to enable communication on a flock of fixed wing MAVs due to its combination of low energy consumption and long range (offering “*a range of over 1 mile at 60 mW”*). For comparisons of technical details of these technologies we refer the reader to the detailed book by Bensky (2019), the MAV-focused review by Zufferey et al. (2013), as well as the earlier comparisons by Lee et al. (2007).

In addition to the technologies discussed above, there is also the possibility of enabling indirect communication via cellular networks. In the near future, 5G networks are expected to make it possible to have a reliable and high data throughput between several MAVs (Campion et al., 2018). Finally, the use of UWB can also gain more relevance in the future, especially because its additional capability to accurately measure the range between MAVs, as discussed in section 5.1, can be very helpful for swarms. One technological challenge is that communication needs power, and while this may be near-negligible for the bigger MAVs, it is not so for the smaller designs (Petricca et al., 2011). From this perspective, the communication-based relative localization discussed in section 5.1, which can also double as a communication device for MAVs, is an interesting solution if one desires a system that can achieve both goals simultaneously. However, using any relative localization approach that relies on communication means that having a stable connection among MAVs is an important requirement, and possibly a safety critical one. Moreover, high messaging rates also become important in order to have a high update rate.

## 6. Swarm-Level Control

We finally arrive at the “swarm” part of this paper. Once we have reliable MAVs that can safely fly in an environment, localize one another, and perhaps even communicate, we can begin to exploit them as a swarm. The complexity of this task stems from the fact that, due to the decentralized nature of the swarm, the local actions that a robot takes can have any number of repercussions at the global level. These cannot be known unless the system is fully observed and optimized for, which the individual robot cannot do.

This section discusses possible approaches to design MAV swarm behaviors. Prominent examples of behaviors are: flocking, formation flight, distributed sensing (e.g., mapping/surveillance), and collaborative transport and object manipulation^10^. Of these, formation flight receives significant attention. It can be useful for several applications, such as surveillance, mapping, or cinematography so as to collaboratively observe a scene (Mademlis et al., 2019). Additionally, it can also be used for collaborative transport (de Marina and Smeur, 2019), and it has even been shown that certain formations lead to energy efficient flight for groups (Weimerskirch et al., 2001). Flocking behaviors bear similar properties to formation flight, but with more “fluid” inter-agent behaviors that allow the swarm to re-organize according to their current neighborhood and the environment. Distributed sensing behaviors may require the swarm to travel in a formation or flock, but may also include behaviors in which the swarm distributes over pre-specified areas (Bähnemann et al., 2017) or disperses (McGuire et al., 2019). Collaborative transport and object manipulations take two forms. The first is that of MAVs individually foraging for different objects and bringing them to base (Bähnemann et al., 2017), the second is that of jointly carrying a load that is too heavy for the individual MAV to carry (Tagliabue et al., 2019). In order to achieve the behaviors above, and others, the MAVs can also engage in a number of more general swarm behaviors, such as distributed task allocation or collective decision making. For all cases, the challenge is to endow the MAVs with a controller that achieves the desired swarm behavior while also avoiding undesired results (Winfield et al., 2005, 2006).

Similarly to the review by Brambilla et al. (2013) (which the reader is referred to for a general overview of swarm robotics and engineering), we divide the design methods in two categories. The first, which we call “manual design methods,” refers to hand-crafted controllers that instigate a particular behavior in the swarm. These are discussed in section 6.1, where we provide an overview of the state of the art for different swarm behaviors. The second, which we refer to as “automatic design methods,” uses machine learning techniques in order to design and/or optimize the controller for an arbitrary goal. This is discussed in section 6.2. We discuss the advantages and disadvantages between the two, from the perspective of designing swarms of MAVs, in section 6.3.

### 6.1. Manual Design Methods

This is the “classical” strategy to control, whereby a swarm designer develops the controllers so as to achieve a desired global behavior. For swarm robotics, we differentiate between two approaches. One approach is to design local behaviors, analyze them, and then manually iterate until the swarm behaves as desired. Another approach is to make mathematical models of the robots and their interactions and then design a suitable controller that comes with a certain proof of convergence. The latter approach has some obvious advantages if one succeeds, but it makes the designer face the full complexity of swarm systems. Hence, such methods typically have limited applicability. For example, in the work of Izzo and Pettazzi (2007), the behavior is limited to only symmetrical formations of limited numbers of agents. The preferred approach is dependent on the swarm behavior that the designer wishes to achieve, under the constraints of the local properties of each MAV.

A large portion of methods focuses on formation control algorithms, whereby the goal is for the MAVs to form and/or keep a tight formation during flight. To hold a formation, the MAVs must hold a relative position or distance between given neighbors, such that they can move as one unit through space. See, for instance, the works of Quintero et al. (2013), Schiano et al. (2016), de Marina et al. (2017), Yuan et al. (2017), and de Marina and Smeur (2019). One advantage of flying in formation for MAV swarms is their predictability during operations. Several methods provide robust controllers with mathematical proofs that the formation can be achieved and maintained during flight. A review dedicated to formation control algorithms for MAVs is provided by Oh et al. (2015). Chung et al. (2018) also discuss different methods.

There are applications for which a rigid formation is sub-optimal, undesired, or unnecessary, and it is better for the MAVs to move through space in a flock. Flocking behaviors were originally synthesized from the motion of animals in nature (Aoki, 1982), and were most famously formalized by Reynolds (1987) with the intent of simulating swarms in computer animations. The behavior is typically characterized by a combination of simple local rules: attraction forces, repulsion forces, heading alignment with neighbors, speed agreement with neighbors. This behavior naturally incorporates collision avoidance via the repulsion rule, and it has also been explored as a means to collectively navigate in an environment with obstacles, whereby the obstacles provide additional repulsion fores (Saska et al., 2014; Saska, 2015). Alternatively, the local rules can also be exploited to achieve formations by making use of equilibrium points between attraction and repulsion forces (Gazi, 2005). Depending on the way in which the rules are used, they can be incorporated into an iterative approach, or they can be made part of a mathematical regime combined with the model of the robot. An early real-world demonstration of distributed flocking was achieved by Hauert et al. (2011) with a swarm of ten fixed wing MAVs. The more recent work by Vásárhelyi et al. (2018) demonstrated outdoor flocking for a swarm of 30 quadrotors.

Concerning behaviors, such as distributed sensing, exploration, or mapping, there are several different types of solutions that have been developed specifically for MAVs. Typically, these are found to vary depending on the nature of the task, requiring the designer to make careful choices on the best algorithm to be used. Bähnemann et al. (2017) and Spurný et al. (2019), aided by GNSS for positioning, divided a search area into multiple regions so that a team of three MAVs could efficiently explore it with a pre-planned trajectory. The recent work of McGuire et al. (2019) demonstrated a swarm of six Crazyflie MAVs performing an autonomous exploration task in an unknown indoor environment. Each MAV acted entirely locally based on a manually designed bug algorithm which enabled exploration as well as homing to a reference beacon.

### 6.2. Automatic Methods for Behavior Design and Optimization

In the last few decades, the increasing power of machine learning methods cannot be denied, with multiple examples in robotics, autonomous driving, smart-homes, and more. Machine learning techniques offer a way to automatically extract the local controller that can fulfill a task, relieving us from the need to design it ourselves. However, the problem shifts to devising algorithms that can efficiently and effectively discover the controllers for us. In this section, we discuss the possibilities based on two primary machine learning approaches in swarm intelligence research: Evolutionary Robotics (ER) and Reinforcement Learning (RL).

#### 6.2.1. Evolutionary Robotics

ER uses the concept of *survival of the fittest* in order to efficiently search through the design space for an effective controller (Nolfi, 2002)^11^. It has been widely adopted in swarm robotics literature in order to evolve local robot controllers that optimize the performance of the swarm with respect to a global, swarm-level objective (Trianni, 2008). ER bypasses the analysis of the relation between the local controllers and the global behavior of the swarm. Instead, it optimizes the controllers “blindly” by means of several evaluations in an evolutionary process, which most often happens in simulation, but can also be performed in the real world (Eiben, 2014). Evolved solutions often exploit the robots' bodies and environment, including the behaviors of other swarm members. Moreover, thanks to the blind optimization, not only the controller can be evolved, but also other factors, such as the communication between robots (Ampatzis et al., 2008). Likewise, ER offers a generic approach to generate swarm controllers of different types, including, but not limited to: neural networks (Trianni et al., 2003; Silva et al., 2015), grammar rules (Ferrante et al., 2013), behavior trees (Scheper et al., 2016; Jones et al., 2018, 2019), and state machines (Francesca et al., 2014). Although neural network architectures can be very powerful, the advantage of the latter methods is that they can be better understood by a designer, which makes it easier to cross the “reality gap” between simulation and the real world when deploying the controllers on the real robots (Jones et al., 2019). Crossing the reality gap is a major challenge in the field of ER and many different approaches have been investigated, also for neural networks. See Scheper (2019) for a more extensive discussion on these methods.

A major challenge for the effective use of ER, especially for swarm robotics, is the design of the fitness functions to be optimized (Francesca and Birattari, 2016). This is usually left to the designer's ability to explicitly define the key elements that indicate the success of a behavior in a measurable and quantitative manner. It is not uncommon to see empirically defined parameters that represent certain desired elements, such as “safety” in the example of Duarte et al. (2016). As task complexity increases, so does the challenge of designing a fitness function. In the worst case, it may become *uninformative* or even *deceptive*, leading the algorithm to not finding the desired behavior (Silva et al., 2016). Different approaches have been proposed to tackle this issue, such as behavioral decomposition or incremental learning (Nelson et al., 2009). The risk with these strategies, however, is that the designer shapes the learning of the task too much, which may lead to sub-optimal performances. As an alternative strategy for learning complex tasks, Lehman and Stanley (2011) proposed novelty search, whereby the fitness is not defined by how well the task is performed, but by how “novel” a behavior is. This can lead to finding more unorthodox solutions, also for swarm robotics (Gomes et al., 2013). Potential drawbacks of this approach are that the search becomes less directed, and that the shaping shifts from defining a fitness function to defining what constitutes a “behavior.”

To conclude, the ER approach applied to swarming has the large advantage that it deals with complexity by actually bypassing it. However, this currently comes at the cost of needing many evaluations involving the simulation of not one but multiple robots, which leads to longer lasting evolutions. An additional problem of simulating a specific number of robots to evolve a swarm behavior is that the evolution may overfit the behaviors not only to the (simulation) environment, but also to the exact number of robots that were used during the evolution. A naive solution is to simulate different swarm sizes over the evolution, but this will take even more simulation time, and in any case, the number of robots will be limited, meaning that scalability is not guaranteed. Recent developments in this domain have seen the introduction of size-agnostic techniques (Coppola et al., 2019). Finally, although there are studies on online evolutionary learning for swarm robotics (Bredeche et al., 2018), online evolutionary strategies have yet to be explored (in practice) for MAVs.

#### 6.2.2. Reinforcement Learning

With RL, a robot is made to learn by trial-and-error from interacting with its environment under a certain reward scheme. This approach teaches the robot an optimal mapping between a state and the action that it should take so as to maximize its final reward (Sutton and Barto, 2018). RL has been widely used in robotics, and it has thus also found its way to swarm robotics (Brambilla et al., 2013). The advantage of RL is that the robots can explore the environment and continuously adapt their behavior. Several techniques have been proposed over the years for multi-agent RL (Busoniu et al., 2008). However, within swarm robotics literature, it has generally received less attention than ER (Brambilla et al., 2013). A main difficulty with this approach is that, from the perspective of the individual robot, being in a swarm is a non-Markovian task, and each robot only has a partial observation of the full global state. A potential issue, for instance, is “state aliasing,” which refers to when multiple states appear to be the same from the perspective of the agent, even though they are not (McCallum, 1997). It has been demonstrated that ER can achieve better solutions for non-Markovian tasks (de Croon et al., 2005).

The solution to use RL with non-Markovian task leads to a Partially Observable Markov Decision Problem (POMDP). In this case, a robot keeps a history of its observations and thus extracts the most likely global state from them. RL can be applied to POMDPs (Ishii et al., 2005), yet features scalability issues (the so called “state explosion”), especially when ported to the swarm domain because the global state of the swarm, which it tries to estimate, can take exponentially many forms (Parsons and Wooldridge, 2002). In recent work, Hüttenrauch et al. (2019) proposed to use mean feature embeddings which encode a mean distribution of the agents. This compression is then invariant to the number of agents in the swarm. Another known difficulty of RL with respect to ER is the credit assignment problem. This refers to the challenge of decomposing the global rewards into local rewards for each robot, as the individual contribution of a single robot to a global task may not always be clearly determined (Brambilla et al., 2013). The credit assignment problem is also manifested over time, as it is difficult to judge which prior action was most conducive.

In short, until now ER appears to be a more appropriate choice for learning control in swarms, as it allows robots to exploit non-Markovian properties of the problem (e.g., the states and behaviors of other robots). However, because of the reality gap, online learning methods may turn out very useful in the future, including RL methods.

### 6.3. Manual vs. Automatic Methods for MAV Swarms

A primary advantage of manual design methods for MAV swarms is that the solutions are generally better understood, given that they have to be designed and programmed manually. The algorithms that are developed can be analyzed, and in certain cases it can even be assessed whether the system will converge to the desired properties and even be resilient to faults (Saldaña et al., 2017; Saulnier et al., 2017). This is a particularly attractive property for MAV applications, where safety and predictability are a primary concern. A second advantage is that they carry a clearer breakdown of the requirements. For these reasons, it is not surprising that, to the best of our knowledge and as confirmed by Chung et al. (2018), most real-world implementations of MAV swarms to date have relied on primarily manually designed swarming algorithms. These advantages have also been acknowledged by the automatic design community, which has brought a general interest in using automatic approach to develop explicit controllers, such as state machines (Francesca et al., 2014, 2015) or behavior trees (Kuckling et al., 2018; Jones et al., 2019). In future work, the use of these methods could lead to a compromise between extracting an understandable controller and exploiting the power of automatic methods.

A challenge of designing an algorithm manually is in the need to ensure that it can work within the limitations of the system. For instance, if using a communication-based ranging relative localization system, the relative location estimate is only observable when both MAVs are moving in such a way that the system is excited (Nguyen et al., 2019). Alternatively, cameras can be limited by the FOV and be forced to keep a reference neighbor in the center (Nägeli et al., 2014). This may be undesirable for the final application of the swarm (e.g., surveillance), since the camera is kept pointing to other MAVs as opposed to interesting features in the environment. Examples, such as these serve to show how a manually designed algorithm can either fail to regard certain elements, or may not exploit the environment optimally so as to best deal with the limitations. An automatic method, on the other hand, could extract a controller that best deals with the limitations, possibly finding solutions that cannot be easily designed manually. For instance, ER studies show that evolved robot controllers can find behaviors that tightly exploit the sensory and motor capabilities of the given robot (Nolfi, 2002)—this is called sensory-motor coordination.

Despite their power, the application of automatic design methods to MAV swarms are relatively few. One of the first steps was done by Hauert et al. (2009) for the purposes of developing a flying communication network. In this case, the authors proposed to reverse engineer the behavior of an evolved neural network and subsequently program a similar behavior manually. This approach provided original and “creative” insights that enabled them to design a viable and flexible behavior. In later work, Szabo (2015) applied evolutionary behavior trees to a team of MAVs for the purposes of collision avoidance, exploiting the increased readability of behavior trees. The MAVs only knew each other's relative distance (not position) as measured by noisy Bluetooth signal strength, yet the evolved behavior was capable of reducing the number of collisions in a cluttered space. The automatically evolved behavior tree was not only simpler (fewer nodes/branches), but also performed better when compared to a manually designed one. Scheper and de Croon (2017) trained a neural network to form a triangle with a team of three MAVs, inspired by a similar task by Izzo et al. (2014). Although not aimed at MAVs, Izzo et al. (2014) had previously shown that an automatic method was able to extract a behavior with which homogeneous agents could self-organize into asymmetric patterns, whereas the previously developed manual approaches for the same system were limited to symmetric patterns (Izzo and Pettazzi, 2007). Scheper and de Croon (2017) additionally showed that evolving a controller at a higher level of abstraction does not necessarily compromise the ability of automatic methods to exploit an environment and sensory-motor relationships, yet helps to reduce the reality gap. The more recent work of Schilling et al. (2019) showed that it's possible to learn a flocking behavior directly from camera images using imitation learning. This was demonstrated in a real world environment with two MAVs. This automatic approach was able to find a viable, collision-free behavior that could also localize neighbors.

The limited amount of works show that this field is still young. The extra challenge comes from the several constraints that flow from the lower levels as well as the additional cost and difficulty of real-world experimentation. Nevertheless, there are arguments to show that automatic methods may eventually provide a way to make the most out of the swarms (Francesca and Birattari, 2016). We expect that in the future, once both MAVs as well as automatic swarming design technologies become more mature, we will begin to see an increase of (experimental) works in this domain.

## 7. Further Challenges and Future Developments to Be Made

### 7.1. Battery Recharging and Scheduling

As already discussed, flight time is a fundamental constraint for MAVs. Swarming can help to expand the flight time of the whole system, such that a portion of MAVs can recharge while others are still in operation. This is subject to two main challenges. The first is the design of the combined MAV + re-charging ecosystem, and the second is the distributed scheduling between drones. Research has already begun on this front, albeit to the best of our knowledge an automated and distributed recharging method for a swarm of MAVs has yet to be demonstrated outside of a controlled environment. Toksoz et al. (2011) and Lee et al. (2015) designed a battery swapping station to quickly exchange batteries on a quadrotor. The advantage of such a system is that the battery can be changed quickly. However, it also requires an intricate design as well as highly accurate landing to ensure that the battery is properly replaced. Instead, a contact-based re-charging station, such as the one proposed by Leonard et al. (2014) offers a simpler system, albeit at the cost of a slower turn-over. The authors investigated its use for a multi-UAV system, whereby the MAVs queued their use of the charging stations via a prioritization function. Using a similar charging system, Mulgaonkar and Kumar (2014) demonstrated a system where three quadrotors take turns to surveil a target region, such that one operates while the other two recharge. Vasile and Belta (2014) and Leahy et al. (2016) proposed formal strategies based on temporal logic constraints to ensure that the MAVs would correctly queue for recharging. However, the experimental efforts focused on the case where only one MAV operates at a given time. Nowadays, commercial charging station are also available (Brommer et al., 2018). This will likely accelerate the research progress. Wireless charging, albeit slower, is also an attractive choice as it softens the requirement on precision landing (Choi et al., 2016; Junaid et al., 2017).

Flight time can also be increased at the MAV design level by designing MAVs with on-board recharging or longer endurance. The capability for long endurance would allow the swarm to be more flexible and take on a more diverse set of missions. One possible method to increase the flight time is to use solar cells. These have mostly been applied to fixed wing designs, such as the “Skysailor” MAV (Noth and Siegwart, 2010), benefiting from efficient flight conditions and large wing areas. It can in fact be shown that the benefit of solar cells begins to have little effect on smaller platforms, due to the reduced surface area available (Bronz et al., 2009). This trend is even more prominent on quadrotors, which have higher energy requirements. As a solution, D'Sa et al. (2016) proposed an MAV design that can alternate between fixed wing and quadrotor mode, such that “*surplus energy collected and stored while in a fixed wing configuration is utilized while in a quadrotor configuration*.” Recently, Goh et al. (2019) demonstrated a fully solar-powered quadrotor. To meet the energy requirements, an area of 4 m^2^ was required together with a reliance on ground-effects, meaning that the MAV was bound to low altitudes. A different solution is to use combustion engines (Zufferey et al., 2013; Nex and Remondino, 2014; Ross, 2014). They benefit from the high-energy density of fuel and can help to provide long endurance flight, although they are typically applied to larger drones in outdoor environments Alternatively, fuel cells have also been explored as a power source for long endurance flight, with increasingly promising results in the recent years (Gong and Verstraete, 2017; De Wagter et al., 2019; Pan et al., 2019).

### 7.2. Swarm-Level Active Fault Detection

Active and decentralized fault detection should also play a fundamental role for the realization of MAV swarms^12^. If not catered to, then there is a risk that the erroneous actions of one MAV hinder the entire swarm (Bjerknes and Winfield, 2013). Winfield and Nembrini (2006) applied the Failure Mode and Effect Analysis (FMEA) methodology to evaluate the reliability of an entire swarm based on its possible failure points. From such studies it can be evaluated whether, and to what extent, local failures can incapacitate the swarm. The question is how such faults can be detected and dealt with during operations. Doing so would create a system that is more robust to failures.

Li and Parker (2007) developed the Sensor Analysis based Fault Detection (SAFDetection). In this approach, a clustering algorithm is used to learn a model of the robots' expected behavior. This model is then used to determine whether the behavior of a robot in the swarm can be considered “normal” (i.e., falls within the learned model), or “abnormal,” in which case a likely fault has been detected. A distributed version of the algorithm has also been developed (Li and Parker, 2009), in which case each robot learns its own behavior model locally and then shares it. This strategy scales better with the size of the swarm, as it parallelizes the clustering computations. The works by Tarapore et al. (2013, 2015a,b) also propose a strategy for normal/abnormal behavior classification by synthesizing the behavior of neighbors within a binary feature vector. In more recent work, Tarapore et al. (2017) proposed the use of a consensus algorithm so that the robots can collectively reach a decision on whether the behavior of a team-member can be considered normal or abnormal. This was also tested on a real robotic system (Tarapore et al., 2019). Qin et al. (2014) provide a review on this active area of research. Bringing these solutions to MAV swarms can largely improve the operational safety of the full system, which is paramount for deployment in the real world.

### 7.3. Controlling and Supervising Swarms of MAVs

A control interface should enable an operator to provide commands to the swarm, such as take-off and landing, the commencement of mission objectives, or the engagement of swarm-wide emergency procedures. All should be done in a direct and intuitive way to minimize the effort by the operator (Fuchs et al., 2014; Dousse et al., 2016). To this end, Nagi et al. (2014) explored the use of a gesture vocabulary which allows a human operator to instruct a team of MAVs. The human operator and the gestures are detected directly by the MAVs using their on-board camera. Thanks to their multiple viewpoints, they are able to discern the operator's commands in a distributed fashion. Tsykunov et al. (2018) explored how to use a haptic glove to control a team of drones as if they were all connected via a spring-damper system. Research has also focused on the development of gesture languages, as in the works of Soto-Gerrero and Ramrez-Torres (2016) and Couture et al. (2018). Virtual reality is also becoming an increasingly popular technology, and is beginning to be applied to the control of MAVs (Tsykunov and Tsetserukou, 2019; Vempati et al., 2019). Besides the above, a less technical, yet highly significant, challenge to overcome on this front is the (understandably) stringent legislation surrounding MAV flight, particularly in outdoor scenarios, often requiring at least one pilot per drone (the specifics vary based on the location) (Vincenzi et al., 2015). We refer the interested reader to Hocraffer and Nam (2017) and the sources therein for a more thorough overview of the challenges and the current technologies for human control of aerial swarms.

## 8. Discussion: How Far Are We From MAV Swarms?

Following the many topics discussed in this paper comes the inevitable question: how far are we from large scale aerial swarms that can cooperatively explore areas, carry heavier objects, and autonomously complete complex tasks without low level human-in-the-loop control? Despite the large amount of research and development that has been done to tackle the topics within this grander scheme, the field of robotics and the field of swarm intelligence are both still relatively young, and there remain advances to be made. In this paper, we discussed how the swarm behavior depends on the constraints set by lower level properties, and vice versa. This interdependency and iterative nature of design means that, if we wish to bring full-fledged MAV swarms to the real world, there must be a mutual understanding between the design levels as to what is required and what can be achieved in reality.

One of the main technologies required to make the leap from flying a single MAV to flying a decentralized swarm is an accurate and reliable intra-swarm relative localization technology. Even for those applications where cooperation is limited and each member in the swarm acts mostly independently, relative localization is still needed to ensure relative collision avoidance, which is a safety-critical requirement. As we have shown throughout this paper, several technologies are currently under exploration and it is still unclear which will prove most reliable and advantageous in the long run. As the choice of these systems very directly shapes the behavior of the swarm, the challenge of designing the swarm behavior needs to be tightly coupled to it, additionally to the way it is coupled to the design of the individual robots. As such, automatic design algorithms of swarm behaviors can provide a way to make the most out of the individual MAVs and their limitations, albeit at the potential cost of relying on less well-understood controllers.

Additionally, the on-going standardization of tools is expected to help the field to reach a new level of maturity (Nedjah and Junior, 2019). Systems, such as ROS (Quigley et al., 2009), Paparazzi (Mueller and Drouin, 2007; Brisset and Hattenberger, 2008), or PX4 (Meier et al., 2015) have now accelerated the process of prototyping and testing on real-world MAVs, and have also made it easier to share hardware/software advancements. Low cost programmable MAVs, such as the Crazyflie are also available, making it more feasible to experiment with large numbers of MAVs. Additionally, dedicated standards, such as MAVLink, which provides communication between software modules, are becoming increasingly popular (Dietrich et al., 2016), and full-stack frameworks have been developed to handle the entire pipeline (Sanchez-Lopez et al., 2016; Millan-Romera et al., 2019). The combination of these systems together with simulators, such as the well-known Gazebo (Koenig and Howard, 2004), ARGoS (Pinciroli et al., 2012), or AirSim (Shah et al., 2018), further help to quickly prototype software in a realistic simulation environment. Combined with models and frameworks, such as hector-quadrotor (Meyer et al., 2012) or RotorS (Furrer et al., 2016), simulation environments can significantly accelerate the development time (Johnson and Mishra, 2002). Mairaj et al. (2019) provides an extensive review of several simulators for this purpose. Dedicated swarm languages, such as Buzz (Pinciroli and Beltrame, 2016) also provide a simpler prototyping framework dedicated to swarm robotics, which can also be applied to MAVs.

Finally, the prominent rise in popularity of MAVs in the last decade has brought about several technology accelerators. MAV focused robotics competitions, such as the Mohamed Bin Z¨ayed International Robotics Challenge (MBZIRC) or the International Micro Air Vehicle (IMAV) competition have now also begun to integrate swarming or multi-robot elements (Pestana et al., 2014; Saska et al., 2016a; Bähnemann et al., 2017; Nieuwenhuisen et al., 2017; Spurný et al., 2019). This pushes researchers to take a technology out of the lab and into unknown environments, thereby increasing their robustness.

## 9. Conclusion

The challenges to solve before we can expect to see swarms of autonomous MAVs are many. They begin at the lowest level, forcing us to think of how the MAV design will impact the swarm behavior, and they end at the highest level, where we must design collective behaviors that best exploit our lower level designs, controllers, and sensors. In the last decade, the field of swarm robotics and MAV design have started to merge more and more, leading to increasingly impressive achievements. To go further, the tight and complex relationship between the low level and the high level needs to be appreciated in order to break into a new era of truly autonomous and distributed swarms of MAVs.

## Author Contributions

This article has been set up based on discussions between all authors. It has been primarily written by MC with the additional help and advice of KM, CD, and GC.

### Conflict of Interest

The authors declare that the research was conducted in the absence of any commercial or financial relationships that could be construed as a potential conflict of interest.

## Footnotes

^1^Of course, flying several MAVs with a centralized controller has its own challenges, which we do not mean to undermine. We only mean that it appears that these methods are at a more mature stage with respect to self-organized approaches, which is the focus of this article.

^2^When we relate this to nature, then low-level control and state-estimation seldom requires large “computational” efforts by the individual animal. Rather, they eventually become second nature (Rasmussen, 1983). The real focus is directed to higher level tasks.

^3^“PMW3901MB Product Datasheet” by PixArt Imaging Inc., June 2017.

^4^See www.st.com/en/imaging-and-photonics-solutions/vl53l0x.html

^5^See www.bitcraze.io/multi-ranger-deck/

^6^The solution by Teixeira et al. (2018) additionally uses communication between the MAVs.

^7^WiFi also operates at other frequency bands. The 5 GHz band, for instance, is typically known to feature a lower interference (Verma et al., 2013). ZigBee can also operate at the 868 and 915 MHz frequency bands (Collotta et al., 2018).

^8^Not to be confused with ZigBee (Faludi, 2010).

^9^Note that Bluetooth Low Energy, a sub-version of the Bluetooth standard, also requires very little power. Tests by Collotta et al. (2018) return that Bluetooth 4.2 and 5.0 have a lower power consumption than ZigBee.

^10^Note that this list not exhaustive. Additionally, we will see that there may also be overlaps between these behaviors. For example, as explored in section 6.1, flocking behaviors may achieve fixed formations under certain equilibria.

^11^Looking at the complexity achieved by natural swarming systems, it also seems intuitive that such complexity could be achieved automatically by mimicking an evolutionary process (Bouffanais, 2016). It is no surprise that a closely related discipline to ER is that of Artificial Life (AL), dedicated to artificially representing life-like processes, albeit with generally more open ended exploratory goals (Bedau, 2003; Trianni, 2014).

^12^We differentiate between fault *detection* and fault *tolerance*. Fault tolerance refers to the ability of the system to be robust to faults. Fault detection refers to the ability of the robots in the swarm to detect issues, and thus possibly also cope with them.
